# The Role of the Interferon Gamma Release Assay in Assessing Recent Tuberculosis Transmission in a Hospital Incident

**DOI:** 10.1371/journal.pone.0020770

**Published:** 2011-06-13

**Authors:** Louise Bradshaw, Elizabeth Davies, Michael Devine, Peter Flanagan, Paul Kelly, Kevin O'Connor, Francis Drobniewski, Vladislav Nikolayevskyy, Ibrahim Abubakar

**Affiliations:** 1 Tuberculosis Section, Respiratory Diseases Department, Health Protection Agency Centre for Infections, London, England; 2 Northern Health and Social Care Trust, Northern Health and Social Care Trust Headquarters, Ballymena, County Antrim, Northern Ireland; 3 Health Protection Agency National Mycobacterium Reference Laboratory, London, England; McGill University, Canada

## Abstract

In 2007, an extensive contact screening investigation into onward transmission of tuberculosis was instigated at a hospital in Northern Ireland following diagnosis of pulmonary multi-drug resistant TB in a healthcare worker. Interferon gamma release assays (IGRAs) were used to test 333 patients and 98 staff. We investigated for evidence of onward transmission and recent infection based on analysis of clinical, demographic and IGRA data. We also described within-patient variability of IGRA results. Among patients and staff, increasing age of patients was the only factor associated with IGRA positivity. Greatest within-subject variability of IU/mL in serially-tested patients/staff was seen in those with a positive IGRA test and this did not correlate with increased exposure to the index case. IGRA positivity being largely explained by increasing age in patients and previous TB contact in staff lends weight to the conclusion that IGRA positivity reflected previous infection rather than recent transmission.

## Introduction

Tuberculosis (TB) is a leading cause of global morbidity and mortality. The global effort to control TB is under immense threat from drug resistance [1]. Since its emergence in the 1990s, multidrug resistant (MDR-TB) - defined as strains resistant to first-line drugs Isoniazid and Rifampicin, has become an important public health problem in many countries. In the UK, while MDR-TB still remains relatively uncommon, accounting for less than 2% of TB cases [2], the diagnosis of such patients leads to extensive investigation to ascertain whether transmission has occurred [3]. Contacts of MDR-TB cases are however, not usually treated for latent infection due to the absence of effective drug treatment [4].

In June 2007, an extensive contact screening investigation into onward transmission of TB was instigated at a hospital in Northern Ireland following the diagnosis of pulmonary MDR-TB in a healthcare worker. Northern Ireland accounted for <1% (n = 66) of total UK cases in 2007; a rate of 3.8 per 100,000 [2]. The proportion of all notified cases that were diagnosed in healthcare workers in Northern Ireland rose from 1% in 1997 to 12% in 2007 [5].

The contact investigation made use of QuantiFERON®-TB Gold In-Tube (Cellestis Ltd, Carnegie, Australia) interferon gamma release assays (IGRAs) to test 333 patients and 98 staff members. Many of the patients screened had a significant co morbidity including several diseases that increase the probability of developing active TB. IGRAs measure *in vitro* interferon gamma production following stimulation of T-cells by challenge from TB antigens produced from the region of difference present in the *Mycobacterium tuberculosis* complex genome, but not the genomes of *Mycobacterium bovis* BCG or the majority of environmental mycobacteria [6].

Based on the observation that the assays measure interferon produced by a subset of effector memory T-cells which are present during current infection [7], [8], it has been hypothesised that IGRA positivity implies recent infection [7]. However, currently there is no evidence that available IGRAs distinguish active from latent infection [9], [8] We hypothesised that if IGRA positivity uniquely represents recent infection, then among a cohort of untreated contacts, a small proportion of IGRA positive immunocompromised individuals will progress to active TB irrespective of their age. If, however, IGRAs largely represent prior exposure then increasing age will correlate with the test positivity. The results of the investigation and two years of follow-up allowed us to investigate: (1) whether there is evidence of onward transmission resulting from this incident and, if so, (2) whether individuals testing IGRA positive are likely to have been recently infected based on analysis of the IGRA results. We also described within-patient variability of IGRA results.

## Methods

### Patient population and assessment

All eligible contacts who were immunocompromised (determined using pre-specified criteria) and housed in the same ward that the HCW was working were contacted for screening. Screening of 333 immunocompromised persons of 1189 in-patients was undertaken on the basis of their susceptibility to progress to active TB and degree of exposure to the index case, determined from shift rotas and admissions data. Pre-specified criteria to determine eligible patients, using a list of conditions causing immuno-deficiency (including: HIV infection, diabetes, chronic renal failure, transplant patients, cancer patients, immunosuppressive therapy - for steroids, over 4 weeks therapy with at least 20 mg daily was used as an approximate guide) and therefore an increased risk of progressing from latent to active disease was used. Screening of patients was carried out by means of one to three QuantiFERON®-TB Gold In-Tube IGRAs, carried out six to eight weeks apart. Laboratory tests were conducted in the Clinical Pathology Accreditation (UK) Ltd-accredited National Reference laboratory in accordance with the manufacturer's (Cellestis Ltd, Carnegie, Australia) recommendations. All tests results have been validated using built-in internal quality control criteria.

### Follow- up

**Figure 1:**
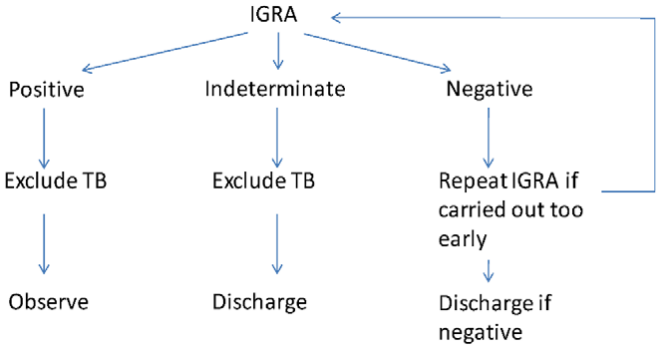
Patient follow-up.

Patients whose initial test was indeterminate but whose second test returned a negative result were regarded as not infected and no further action was taken (see Figure 1). Of patients who were indeterminate on two tests six to eight weeks apart, or negative on the first test and indeterminate on a second, those with a previous history of TB; those with a visible healed tissue on chest x-ray; and those with old calcified disease on chest x-ray were excluded from active further follow-up. The case notes and available radiology were reviewed for all patients with a positive IGRA blood test, and they were followed-up as though they had recent infection. They were kept under out-patient department review for over two years since last possible contact with the index case (the period over which they are at greatest risk of progression to active disease) ending August 2009, after which they were discharged with letters sent to their General Practitioners. Over this period, chest x-rays were taken at intervals of: 6–8 months; 16–18 months; and at two years. Provision was made for review on request if they developed symptoms of TB. No chemoprophylaxis was offered.

### Staff

Ninety-eight staff identified from shift rotas as having been in contact with the index case were screened by single IGRA and, where aged less than 36 years with no history of BCG, a Mantoux tuberculin skin test (TST).

### Long term surveillance

No further screening was scheduled to be undertaken, subject to review if any active cases of TB developed among the cohort. The regional Centre for Disease Surveillance and Control was requested to notify the hospital trust, should any case of MDR-TB be identified in Northern Ireland, in order to investigate possible links with the hospital incident. In addition, patients excluded from active follow-up were searched for in the national MDR-TB register to determine if any progressed to active TB.

### Analysis

Clinical and demographic details were collected for staff and patients by the incident management team as part of the screening exercise, and retrospectively using staff records, interviews and patient charts. Proportions were compared using Pearson's chi-square and means using the t-test. Univariate and multivariable logistic regression was carried out and odds ratios and 95% confidence intervals presented. Analysis was carried out using STATA version 11.

### Ethics

The Health Protection Agency (HPA) has the right under Section 60 of the NHS Act 2006 and approval from the National Information Governance Board to hold and analyse national surveillance data for the purposes of protecting public health and for infectious disease surveillance, as such there was no requirement to approach a separate ethics committee.

## Results

**Figure 2:**
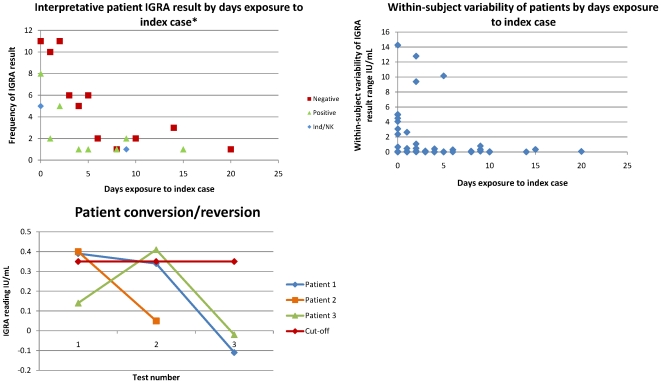
Study IGRA results. A, Interpretative patient IGRA result by days exposure to index case. *Serially IGRA-tested patients only. B, Within-subject variability of patients by days exposure to index case. C, Patient conversion/reversion.

Among 333 patients, 30 (9.01%) had a positive IGRA test, and 41 (12.31%) had an indeterminate result, while seven of 98 staff tested were positive and one had an indeterminate test. The median age of patients was 65 years (inter-quartile range 48–76) and 54.7% were male. Staff had a lower median age (41 years, inter-quartile range 33–46) and 8.16% were male. Only one patient converted from IGRA negative to IGRA positive, however, they later reverted (patient 3 in Figure 2c: conversion plus known exposure to the index case could potentially be interpreted as indicative of recent transmission). This patient had 4 days potential exposure to the index case. Patients 1 and 2 (also Figure 2c, with 2 days and 15 days exposure respectively, and positive interpretative results) also reverted. No patient or staff member has been identified with active TB up to 1 June 2010 which is three years after last possible exposure to the index case.

### Association between patient characteristics and latent infection

Three hundred and thirty-three patients were identified for contact screening (Table 1). This population had a roughly equal gender split and approximately half (53%) had a co morbidity/use of immunosuppressive or steroid drugs. Five patients were known to have a history of TB and one had a family history of TB; two of these tested IGRA positive. Days of exposure to the index case ranged from 0–36 days (median = 1). Table 1 shows demographic and clinical characteristics among staff and patients, and association with IGRA results.

**Table 1:** Demographic and clinical characteristics of staff (n = 98) and patients (n = 333).

Characteristic	IGRA positive	IGRA negative
	n (%)	n (%)
Staff		
Characteristic		
Total	7	90
No. (%) male	1 (14.3)	7 (7.8)
Age (yr)		
Median	45	41
IQR	32.5–52	33–46
Country of birth [No. (%)]		
UK	6 (85.7)	79 (87.8)
Non-UK (Canada, Germany, Philippines, Republic of Ireland)	1 (14.3)	10 (11.1)
Unknown	0 (0)	1 (1.1)
BCG vaccination status [No. (%)]		
Vaccinated	2 (28.6)	76 (84.4)
Unknown	0	6 (6.7)
TST (where age < = 35) [No. (%)]		
Total	2	32
Positive (> = 15 mm induration)	0	6 (18.8)
Unknown	0	2 (6.25)
Co-morbidities [No. (%)]		
Immunocompromised	0	0
HIV	0	0
Diabetes	1 (14.3)	0
Previous TB contact	3 (42.9)	16 (17.8)
Patients		
Characteristic	IGRA positive	IGRA negative
Total	30	262
No. (%) male	17 (56.7)	145 (55.3)
Age (yr)		
Median	70.5	64
IQR	65.5–74	45–77
Co-morbidities [No. (%)]		
Diabetes	7 (23.3)	64 (24.4)
Cancer	8 (26.7)	69 (26.3)
Immunosuppressive drugs/chemotherapy*	1 (3.3)	42 (16)
Steroids	3 (10)	41 (15.6)
Unknown	1 (3.3)	8 (3.1)
Any co-morbidity	16 (53.3)	165 (63)

Table 2 shows that exposure to the index case did not appear to increase the likelihood of testing positive (p = 0.293). Increasing age was the only variable which carried significant risk of testing IGRA positive (OR 4.12, 95% confidence interval 1.63–10.42, p = 0.003), which was further supported by a test for trend (P = <0.0005). No other factors analysed were significant. Age remained the only significant variable (OR 4.74, 95% confidence interval 1.84–12.25, p = 0.001) after fully adjusting for gender, days potential exposure to the index case and whether or not the patients were immunocompromised through multivariate analysis.

**Table 2:** Odds ratios and p-values for selected demographic and clinical factors of patients with a determinate interpretative IGRA results (n = 292).

Characteristic		IGRA	OR (95% CI)
		Positive n/N (%) or mean (IQR)	
Days of exposure (SD)	Positive	3.93 (4.75)	1.04 (0.97–1.11)
	Negative*	2.95 (3)	
Gender	Male	17/162 (10.49)	1.01 (0.47–2.16)
	Female	13/130 (10)	
Age group	0–64	6/139 (4.32)	
	65–74	24/153 (15.69)	4.12 (1.63–10.42)
Steroids	Yes	3/44 (6.82)	0.60 (0.17–2.07)
	No	26/239 (10.88)	
Diabetes	Yes	7/71 (9.86)	0.94 (0.38–2.30)
	No	22/211 (10.43)	
Cancer	Yes	8/77 (10.39)	1.02 (0.43–2.41)
	No	21/206(10.19)	
Immunosuppressive drugs/chemotherapy**	Yes	1/43 (2.33)	0.18 (0.02–1.36)
	No	28/240 (11.67)	

*Where known (4 values missing).

**Anti-cancer chemotherapy within 4 weeks of exposure to index case or ongoing chemotherapy only.

### Association between staff characteristics and latent infection

Table 1 shows the characteristics of staff by IGRA result. The majority (6/7) of IGRA-positive staff were UK-born, and all were from Western European countries of low national TB burden (the UK and Germany). Forty-three percent (3/7) of the IGRA-positive staff were known to have had previous contact with a TB case compared to the 18% of staff who tested negative, however this was not statistically significant (OR 3.33, CI 0 .68–16.36, p = 0.139). Two staff aged 35 or under with an overall positive IGRA result did not have a concordant TST result; both tested negative. Among staff members who had had BCG, vaccination at school, age was strongly associated with testing negative (OR 0.08, CI 0.08–0.53, p = 0.001). Comparison of the proportions of staff testing positive with or without having had TST administered as part of their screening suggest there was no significant difference in this case between the two groups (p = 0.194). This indicates the absence of boosting of IGRA results by TST.

### Within-patient variability

123 staff and patients were serially IGRA tested (38 staff were tested twice and, of 85 patients, 73 were tested twice and 12 were tested three times). Due to small numbers, the two cohorts were amalgamated. Most staff were Northern Ireland-born so were likely to have had similar low exposure to TB as the patients (though we acknowledge that, due to their profession, they may be at slightly elevated risk of exposure). Though the median within-subject variability was low (0.03 IU/mL), a wide range (0–14.26 IU/mL) was observed. Greatest fluctuation was seen in those who had at least one positive result and were, therefore, assigned an overall interpretative result of testing positive for latent TB infection. Figure 2a shows the data for patients who had multiple tests.

There were three serially-tested patients given an overall interpretive value of being IGRA-positive who demonstrated reversion or conversion-reversion (Figure 2c). Patient 1 had 2 days recorded exposure to the index case; Patient 2 had 15 days exposure; and Patient 3 had 4 days exposure. All three positive test results were close to the 0.35 IU/mL cut-off value and were followed by a much lower reading.

## Discussion

The study found that, among patients, increasing age was the only factor associated with IGRA positivity. No other variables were significantly associated with IGRA results among patients or staff. No cases of active TB were reported despite two years of active monitoring and screening of IGRA positive persons and passive follow-up of an immunocompromised population. The greatest within-subject variability of IGRA readings was seen in those with a positive test and this did not correlate with increased exposure to the index case.

The observed association with increasing age as the only independent risk factor in the multivariable model and the lack of progression to active disease amongst the patient cohort suggests that remote exposure, when TB was more prevalent in Northern Ireland is a likely explanation. For example, between 1950 and 1954 an average of 1732 cases were notified in Northern Ireland [10], compared to 66 notifications in 2007 [2]. An alternative explanation is that IGRA positivity was influenced by place of birth and ethnic origin, variables not collected in this investigation because the patient population was all white and predominantly UK-born. Advancing age also increases the likelihood of exposure to unpasteurised dairy produce (in those born pre-1960) and environmental mycobacteria, some of which cross-react with IGRA tests (*Mycobacterium kansasii*, *Mycobacterium szulgai* and *Mycobacterium marinum*), despite the high specificity of IGRAs for the *Mycobacterium tuberculosis* complex. Furthermore, the lack of association between IGRA positivity and exposure to the index case is consistent with this observation.

A review on the role of IGRAs in healthcare workers found poor levels of concordance between IGRA and TST results unless a study is being undertaken in a country of high TB incidence, but that IGRAs correlated better with TB exposure [11]. There were two staff members aged less than 36 years, with a positive IGRA and negative TST result, of which one had had BCG. TST administration has in some cases been observed to have a boosting effect on subsequent IGRA tests [12], however, no significant difference was found in IGRA results between TST positive and TST negative individuals, where a TST result was known. It has been proposed that many TST negative, IGRA positive results may be attributed to the individual having been remotely exposed to TB many years previously, hence the waning TST response [9]. Despite a lack of statistical significance, among staff previous TB contact seems a compelling explanation for the majority of positive interpretative IGRA results; 42.9% had had a known previous contact in this group compared to 17.8% of those testing negative. Of course, the true numbers of staff (and patients) who have come into contact with humans/animals with tuberculosis is unknown. Swindells *et al.*, found that positive QFT IGRA results in healthcare workers were generally associated with length of time in employment, or profession, used as a proxy measure of their increased likelihood of occupational exposure to TB [11].

School-age vaccination with BCG was found to be significantly associated with testing IGRA negative. Under the school vaccination programme, children are TST screened for current latent TB infection. Those testing positive, and therefore previously infected, are not vaccinated and those testing negative were given BCG. Two of seven staff who tested positive had had school vaccination, the rest were never vaccinated and were therefore likely to have had prior exposure to TB.

In total there were 37 staff/patients who tested IGRA positive. Forty-six percent of these were immunocompromised. Due to the MDR-TB status of the index case none of these were placed on treatment. As a conservative estimate, we might expect 5% of the cohort (2/37 individuals) to progress to active disease if they had been recently infected. After two years of follow-up no case of active disease has been reported further supporting hypothesis that IGRA positive results, at least in this cohort, reflects previous exposure to mycobacteria.

At present data is lacking on the amount of variation that might be expected where individuals are repeatedly IGRA tested, however, significant variation has been reported [12], [13]. This has ramifications for interpretation of single IGRA values (especially those close to the cut-off point) and for thresholds of reversion and conversion [12]. In this dataset, the wide range of IGRA readings derived from serially-tested individuals and the fact that those individuals with a positive IGRA result show the greatest variation makes identification of true-positive results problematic. Results of our study support previous limited evidence of greater variability within positive test results [7]. The changes observed, including reversions in three patients, could be explained by within-subject non-specific biological variability around the cut-off point rather than genuine changes of infection status. These findings are congruent with a recent systematic review on use of IGRAs in screening of healthcare workers, where, of 10 studies using IGRAs for serial testing, all showed great variation in conversion and reversion rates, confounded by a lack of data on optimal cut-off points for serial testing [13].

Most limitations of this study reflect the difficulties involved in carrying out a high-profile and large-scale contact screening investigation based upon a dynamic population. For instance, the collection of duration of exposure was not done prospectively and despite the use of shift duty hours of staff and patient hospital admission dates as a guide, there is likely to be some misclassification. Variables such as previous TB contact rely on recall by the staff or patients and are therefore subject to bias. Other limitations include completeness of medical and admissions records. The small number of IGRA positive individuals among staff also limited the ability of this study to detect factors associated with latent infection.

The absence of TB cases in this cohort and the association with increasing age, would suggest that the universal treatment of casual contacts with a positive IGRA result, especially among older contacts may not be appropriate. These individuals could have acquired latent TB infection when they were younger and adverse effects of chemoprophylaxis are likely to be more common.

Currently, where the index case is not MDR, it is considered unacceptable to disregard a positive IGRA result and consideration is given to treating the patient for latent TB [14]. Furthermore, the significant variability among those testing positive in this contact screening exercise casts doubt on whether single positive IGRA results near the cut-off point or, particularly, positive results followed by reversion, should be considered true-positives. Further predictive value studies with serial testing of participants are needed to investigate this. It has recently been hypothesised that patients remaining persistently positive or that show stable conversion to positive following serial testing will be more likely to progress to active TB [15].

Transmission events from the index case to other staff members or patients are unlikely. No cases of active disease have been reported over two years since the contact screening exercise took place. Each cohort screened had one compelling explanatory variable: age in patients and previous contact in staff which, in this dataset, lends weight to the conclusion that IGRA positivity can not be used as an indicator of recent transmission. Widest variation was seen in IGRA readings from individuals who had multiple readings. Fluctuation and reversion from positive results close to the cut-off point means that positive results should at least be interpreted with caution.
